# Anthricin-induced hyperactive proteasome and its molecular mechanism

**DOI:** 10.1016/j.bbrep.2024.101830

**Published:** 2024-09-23

**Authors:** Kotaro Sakamoto, Runa Fujimoto, Erina Kamiyama-Ando, Takatsugu Hirokawa

**Affiliations:** a Research & Development Department, Ichimaru Pharcos Company Limited, 318-1 Asagi, Motosu, 501-0475 Gifu, Japan; b Division of Biomedical Science, Institute of Medicine, University of Tsukuba, 1-1-1 Tennodai, 305-8575 Tsukuba, Japan; c Transborder Medical Research Center, University of Tsukuba, 1-1-1 Tennodai, 305-8575 Tsukuba, Japan

**Keywords:** Amino acid, anthricin, Deoxypodophyllotoxin, molecular dynamics, protein degradation

## Abstract

Recently, targeted protein degradation has attracted increasing interest as a new drug discovery approach. This method aims to control the function of drug targets by inducing their degradation through protein degradation systems such as the proteasome. Concurrently, compounds that enhance proteasome activity have also garnered attention. In 2023, we reported that anthricin (also known as 4-deoxypodophyllotoxin), a natural product that belongs to the lignan family, enhances proteasome activity. However, whether this enhancement was because of increased proteasome expression or improved proteasome function remains unclear. In this study, we investigated the structure–activity relationship of anthricin and its analogs in enhancing proteasome activity, the effects of anthricin on proteasome-related gene expression, and the direct binding between anthricin and the proteasome using pull-down assay. Moreover, we assessed the interaction between anthricin and the proteasome using molecular dynamics (MD) simulations. The results showed that anthricin does not induce proteasome-related gene expression, but instead binds to the β-subunit of the proteasome, bringing the side chains of three amino acid residues (Thr^1^, Asp^17^, and Lys^33^) at the catalytic site closer together, thereby inducing a hyperactive state. To the best of our knowledge, this study is the first to suggest the mechanism of proteasome activity enhancement by anthricin at the molecular level. The findings could contribute to the development of new chemotypes to enhance the effects of targeted protein degraders by regulating proteasome activity.

## Highlights

• The natural product anthricin, belonging to the lignan family, activates chymotrypsin-like, caspase-like, and trypsin-like proteasomal activities.
• Anthricin does not induce gene expression of the proteasome.
• Pull-down assay demonstrated that antricin directly interacts with the proteasome.
• MD simulation elucidated that anthricin brings the side chains of Thr^1^, Asp^17^ and Lys^33^, inducing a hyperactive proteasome.

## 1 Introduction

The proteasome is a protease complex comprising multiple subunits for degrading proteins that have completed their roles or have become dysfunctional within the cell [1]. After degradation, amino acids are produced, which are then used for the synthesis of new proteins, thereby maintaining cellular homeostasis. In cancer, proteasome activity is often upregulated, which plays a crucial role in tumor growth and survival [2]. Consequently, many proteasome inhibitors, including bortezomib, have been studied and marketed as anticancer drugs [3]. These inhibitors induce denatured protein accumulation in the cell by inhibiting proteasome activity, leading to subsequent antitumor effects through impaired cell proliferation and increased inflammation. Conversely, decreased proteasome activity in normal tissues of the central nervous system is associated with neurodegenerative diseases including Alzheimer's and Parkinson's diseases [4]. Proteasome activity decreases with age. For example, by the age of 40s, proteasome activity in normal human dermal fibroblasts has been reported to decrease to half that of individuals in their 20s [5]. Therefore, the development of proteasome activators as therapeutic drugs for neurodegenerative diseases is being actively pursued [6].

Recently, a novel drug development approach that utilizes the protein degradation function of the proteasome has attracted considerable attention as protein degraders [7]. Conventional drug development approaches aim to control the function of target proteins by directly binding them with small molecules or peptides [[8], [9], [10]]. However, developing drugs for undruggable target proteins that are not enzymes or lack binding sites for ligands remains challenging. Protein degraders work by forcibly adding a tag called ubiquitin to target proteins, which is recognized by the proteasome for degradation. This method is actively being developed as an effective drug development approach even for undruggable target proteins. Moreover, proteasome activators are gaining attention for enhancing the effects of protein degraders, showing promise as a valuable combination in drug development.

**Fig. 1:**
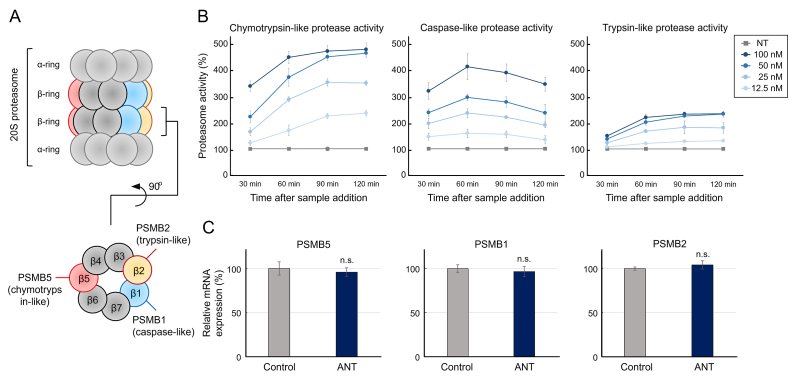
Concentration- and time-dependent effects of anthricin on enhancing intracellular proteasome activity and its influence on proteasome-related gene expression (A) Schematic overview of the 20S proteasome structure. The β1, β2, and β5 subunits possess catalytic sites necessary for protein degradation, which are responsible for caspase-like, trypsin-like, and chymotrypsin-like activities, respectively. (B) Time-dependent effects of anthricin on proteasome activity. Anthricin was added to HEK293T cells, and the proteasome activity was evaluated at 30, 60, 90, and 120 min using the Proteasome-Glo Cell-Based Assay (n = 4, mean ± standard error of the mean [SEM]). The relative values are shown, and the proteasome activity at each time point in the non-treated control group is set to 100 %. (C) Expression of proteasome-related genes with or without anthricin. Anthricin (100 nM) was added to HEK293T cells, and the mRNA expression levels of the β1 subunit (PSMB1), β2 subunit (PSMB2), and β5 subunit (PSMB5) were evaluated after 60 min (n = 4, mean ± SEM). The relative values are shown, and the expression levels of proteasome-related genes at the same time point in the non-treated control group are set to 100 %. ANT, anthricin.

Compared with proteasome inhibitors, there are a limited number of proteasome activators, including natural products such as betulinic acid [11] and ursolic acid [12] and synthetic compounds such as chlorpromazine [13] and MK-886 [14]. The molecular mechanisms of proteasome activation can be broadly categorized into two types: gate opener, which opens the proteasome and promotes protein intake, and allosteric stimulant, which enhances substrate binding and degradation at the catalytic site of the proteasome; however, both of these mechanisms lead to hyperactive states of the proteasome [15]. The proteasome structure can be broadly categorized into two types. One is the 20S proteasome, a hollow barrel-shaped complex with protease activity, which is mainly focused in this study. The other is the 26S proteasome, which is formed by the association of the 19S complex at both ends or one side of the 20S proteasome. The 20S proteasome comprises 14 types, totaling 28 subunits, arranged in four stacked rings. The outer two α-rings function as gates controlling substrate intake and degradation product ejection, whereas the inner two β-rings contain catalytic sites showing chymotrypsin-like (β5), caspase-like (β1), and trypsin-like (β2) activities (Fig. 1A). These β-subunits cannot be catalytically active alone; thus, the formation of substrate binding sites through interactions between β1-7 is important [16].

In 2023, we reported that anthricin, a natural compound belonging to the lignan family that is characteristic of Cupressaceae species such as *Juniperus communis* L., enhances proteasome activity [17]. However, the relationship between anthricin and the proteasome and whether proteasome activation by anthricin was because of the upregulation of proteasome expression or the enhancement of proteasome function remain unknown. In this study, we evaluated the effects of several compounds belonging to the lignan family, including anthricin, on chymotrypsin-like, caspase-like, and trypsin-like proteasomal activities and the effects of anthricin on the gene expression of β-subunits. Furthermore, we validated the interaction of anthricin with the 20S proteasome using pull-down assays and inferred the molecular mechanism of interaction with the proteasome using molecular dynamics (MD) simulations. Our results demonstrated for the first time that anthricin is an allosteric stimulant-type molecule that leads the proteasome into a hyperactive state.

## 2 Materials and methods

### 2.1 Evaluation of intracellular proteasome activity

HEK293T cells (632180, Takara Bio, Shiga, Japan) were incubated in Dulbecco's Modified Eagle Medium with 10 % fetal bovine serum. The cells were seeded in 96-well white tissue culture plates (10,000 cells/well). After 16 h of culture at 37 °C in a CO_2_ incubator, anthricin (CAS No. 19186-35-7; 161–20901, FUJIFILM Wako, Osaka, Japan), podophyllotoxin (CAS No. 518-28-5), 4′-demethylpodophyllotoxin (CAS No. 40505-27-9; CFN90483, ChemFaces, Bukan, China), podophyllotoxin-4-*O*-glucoside (CAS No. 16481-54-2; TRC-P681010, LGC Standards, London, England), yatein (CAS No. 40456-50-6; 29766, Cayman, MI, USA), and (−)-hinokinin (CAS No. 26543-89-5; CFN92255, ChemFaces) were added to the wells. After further incubation (30, 60, 90, or 120 min), the culture supernatant was removed, and the wells were washed once with Dulbecco's phosphate-buffered saline (D-PBS) (043–29791, FUJIFILM Wako). The proteasome activity was estimated using the Proteasome-Glo Cell-Based Assay (G8660, Promega, WI, USA) in accordance with the manufacturer's instructions. Luminescence was detected using a Spectra Max i3 system (Molecular Devices, San Jose, CA, USA).

### 2.2 Measurement of the mRNA expression of proteasome-related genes

Total RNA was extracted from HEK293T cells treated with anthricin (100 nM) for 60 min using the RNeasy Mini Kit (74104, Qiagen, CA, USA) in accordance with the manufacturer's instructions. The quality and quantity of total RNA were determined using the NanoDrop 2000c Spectrophotometer (Thermo Fisher Scientific, MA, USA). Total RNA was reverse transcribed to complementary DNA using the PrimeScript RT Reagent Kit (RR037A, Takara Bio). Relative semi-quantitative real-time polymerase chain reaction (PCR) was conducted in a Thermal Cycler Dice Real-Time System TP800 (GE Healthcare, Buckinghamshire, UK) with the SYBR Premix Ex *Taq*II system (RR041A, Takara Bio) in accordance with the manufacturer's instructions. The thermal cycling conditions were as follows: 30 s at 95 °C, followed by 40 cycles of two-step PCR at 95 °C for 5 s and 60 °C for 30 s and a single cycle of dissociation steps at 95 °C for 15 s, 60 °C for 30 s, and 95 °C for 15 s. Ribosomal protein S18 (RPS18) mRNA was used as the control. The primer set ID for PSMB1 (GenBank Acc. NM_002793.4), PSMB2 (GenBank Acc. NM_001199779.2), and PSMB5 (GenBank Acc. NM_002797.5) are HA352879, HA328427, and HA327634, respectively (Takara Bio). The delta–delta CT method was used to compare mRNA expression in different experimental groups.

### 2.3 Pull-down assay

Human erythrocyte-derived 20S proteasome protein (E−360-050) and Biotin-PEG4-NHS (29114) were purchased from R&D Systems Inc. (MN, USA) and Cyman, respectively. The proteasome protein (150 μg, about 250 pmol) was incubated with Biotin-PEG4-NHS (5000 pmol) for 30 min at room temperature for biotin labeling. Then, the biotinylated proteasome was captured by Magnosphere MS300/Streptavidin (1 mL) (5325, Takara Bio) in 0.1 % BSA containing D-PBS. Anthricin (20,000 pmol; final concentration 20 μM) was added to the solution and incubated for 30 min at room temperature. The beads were washed thrice with D-PBS (500 μL), and bound anthricin was eluted with 100 % methanol (200 μL). As a control, the same work was performed with beads that were not captured with biotinylated proteasomes. The anthricin concentration in the eluent was determined by liquid chromatography with tandem mass spectrometry (LC-MS/MS) using the following conditions: Eluted anthricin was further 10-fold diluted by 100 % methanol and analyzed by LC-MS/MS using a Nexera XR HPLC system coupled with an MS-8050 triple quadrupole mass spectrometer (both from Shimadzu Corporation, Kyoto, Japan). Anthricin was ionized using the DUIS method (electrospray ionization [ESI]/atmospheric pressure chemical ionization [APCI]), which combines simultaneous ESI and APCI operating in the positive mode. The instrument conditions were as follows: interface temperature, 300 °C; heat block temperature, 400 °C; desolvation line temperature, 250 °C; nebulizing gas (nitrogen) flow, 2 L/min; heating gas flow, 10 L/min; and drying gas flow, 10 L/min. Data were acquired in the selected ion monitoring mode (*m*/*z* 399.20). Chromatographic separation was performed on a Unison UK-C18 column (3.0 × 150 mm, 3 μm; Imtakt Corporation, Kyoto, Japan). The column temperature was maintained at 40 °C. The mobile phase comprised methanol and 0.1 % formic acid in water (57:43, v/v) at a flow rate of 0.4 mL/min. The calibration curve was prepared using anthricin (FUJIFILM Wako Pure Chemical Corporation, Osaka, Japan) and was linear over the concentration range of 50–10,000 nM (R^2^ ≥ 0.9996).

### 2.4 MD simulations

The initial structure of proteasome β-ring, which consists of a central two-layer component (composed of 17 chains: 1, 2, H, I, J, K, L, M, N, V, W, X, Y and Z chains) involved in six K-7174 binding was extracted from the crystal structure of 20s proteasome with novel inhibitor K-7174 (PDB-ID: 4EU2) [18]. The anthricin bound proteasome β-ring model was constructed by docking calculations. Six anthricin molecules were docked using six K-1714 molecular binding sites as reference position. After creating six grid boxes, docking calculations were performed in Glide SP mode [19,20] with constraints for the overlap of the common substructures of K-7174 and anthricin, and the structure with the best score in each of the six locations was used as the initial structure for the MD calculation. Apo structure of proteasome β-ring was also set up by removing K-7174. MD simulations of apo and anthricin bound proteasome β-ring structures were carried out using the gDesmond36 ver. 5.7 with the OPLS3e force field [21]. The initial model structures were refined using the Protein Preparation Wizard in Maestro and placed into SPC water molecules solvated with 0.15 M NaCl. After minimization and relaxation of the model, the production MD phase was performed for five independent 200 ns simulations with different initial velocities in an isothermal-isobaric (NPT) ensemble at 300 K and 1 bar using a Nose–Hoover thermostat. Long-range electrostatic interactions were computed using the Smooth Particle Mesh Ewald method. All system setups were performed using Maestro. Trajectory coordinates were recorded every 10 ps. The obtained trajectory was processed utilizing the AmberTools for the calculations of the distances between the side chains of Thr^1^, Asp^17^, and Lys^33^ at the catalytic sites of the β1, β2, and β5 subunits during the 1 μs (5 x 200) simulation.

## 3 Results

### 3.1 Anthricin enhances intracellular proteasome activity in a concentration- and time-dependent manner but does not affect proteasome-related gene expression

Our previous research showed that the chymotrypsin-like activity of the proteasome in HEK293T cells treated with anthricin increased after 2 h [17]. However, the effects over a shorter period: the proteasome activating effect continued until 4 h after sample addition but diminished after 8 h and returned to a steady state after 24 h [17]. In addition, the effects on caspase-like and trypsin-like activities had not been confirmed. Therefore, we evaluated the chymotrypsin-like, caspase-like, and trypsin-like activities at 30, 60, 90, and 120 min after the addition of anthricin to HEK293T cells (Fig. 1B). The chymotrypsin-like activity clearly increased 30 min after the addition of anthricin and plateaued between 90 min and 120 min. When 100 nM anthricin was added, the maximum chymotrypsin-like activity increased to nearly 500 % compared with that of the control group set to 100 %. The caspase-like activity also clearly increased 30 min after the addition of anthricin, with the activity peaking at 60 min and gradually returning to a steady state by 120 min. When 100 nM anthricin was added, the maximum caspase-like activity was about 400 % compared with that of the control group set to 100 %. The trypsin-like activity also clearly increased 30 min after the addition of anthricin, but the degree of increase was more moderate compared with that of chymotrypsin-like and caspase-like activities. The trypsin-like activity plateaued between 60 min and 120 min. When 100 nM anthricin was added, the maximum trypsin-like activity was about 200 % compared with that of the control group set to 100 %. These results indicate that anthricin enhances chymotrypsin-like, caspase-like, and trypsin-like activities of the proteasome in a concentration- and time-dependent manner, especially the chymotrypsin-like activity.

**Fig. 2:**
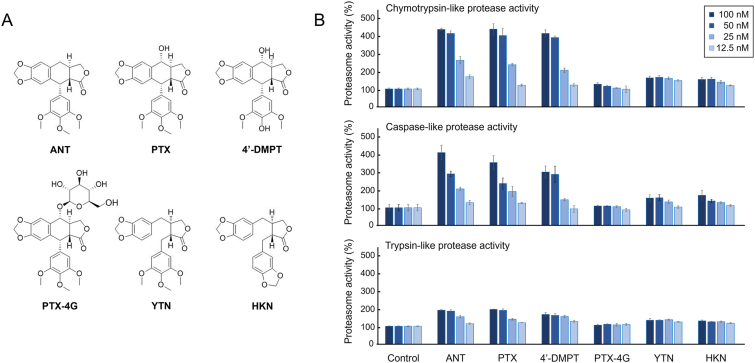
Structure–activity relationships of anthricin and its analogs with proteasome activity (A) Chemical structure of anthricin (ANT), podophyllotoxin (PTX), 4′-demethylpodophyllotoxin (4′-DMPT), podophyllotoxin-4-*O*-glucoside (PTX-4G), yatein (YTN), and hinokinin (HKN). (B) Enhanced proteasome activity of each compound. Each compound was added to HEK293T cells, and the proteasome activity at 60 min was evaluated using the Proteasome-Glo Cell-Based Assay (n = 4, mean ± standard error of the mean). The relative values are shown, and the proteasome activity in the non-treated control group is set to 100 %.

We evaluated the mRNA expression levels of the β5 subunit (PSMB5, responsible for chymotrypsin-like activity), β1 subunit (PSMB1, responsible for caspase-like activity), and β2 subunit (PSMB2, responsible for trypsin-like activity) 60 min after the addition of anthricin to determine whether the enhancement of proteasome activity is qualitative because of improved proteasome function or quantitative because of an increase in the proteasome number (Fig. 1C). The results showed no significant changes in the mRNA expression levels of any of the β-subunits.

**Fig. 3:**
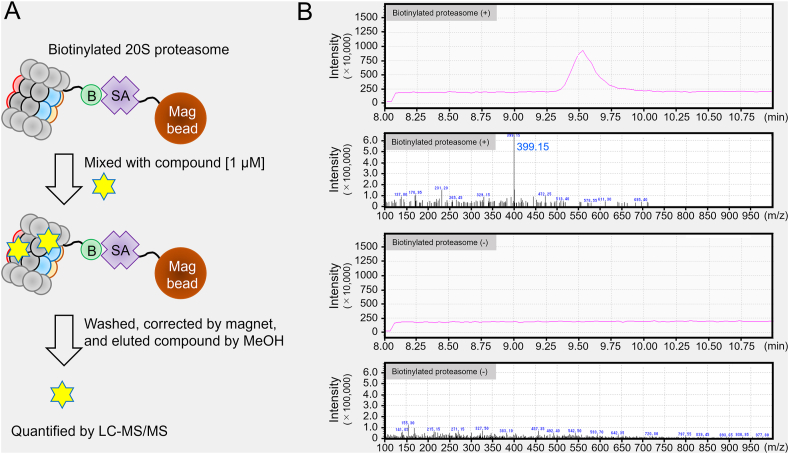
Verification of the interaction between anthricin and 20S proteasome using pull-down assay (A) Schematic of pull-down assay. Biotinylated human erythrocyte-derived 20S proteasome (250 pmol) was mixed with streptavidin (SA) magnetic beads for binding and then reacted with anthricin (20 μM, 20,000 pmol). After the beads were washed, the proteasome was denatured with methanol (200 μL) to elute the bound anthricin (molecular weight = 398.41 g/mol). (B) The amount of anthricin in the recovered methanol solution was quantified using liquid chromatography with tandem mass spectrometry.

The fact that (1) the proteasome activating effect begins to return to a steady state after 8 h [17], (2) the proteasome activity was enhanced within 30 min after the addition of anthricin (Fig. 1B), and (3) there were no changes in the expression level of proteasome-related genes within 60 min (Fig. 1C) strongly suggests that anthricin directly acts on the proteasome and improves its function.

### 3.2 C-ring opening and glycosylation of anthricin greatly attenuate its ability to activate the proteasome

To investigate the structure–activity relationship of anthricin and its proteasome activation ability, we evaluated the effects on the chymotrypsin-like, caspase-like, and trypsin-like activities of five additional compounds: (1) podophyllotoxin with a hydroxyl group added at the 4-position of anthricin, (2) 4′-demethylpodophyllotoxin with the 4′-position being demethylated, (3) podophyllotoxin-4-*O*-glucoside with a glycosylated hydroxyl group at the 4-position, (4) yatein with an opened C-ring on anthricin, and (5) hinokinin with a cyclized methoxy group on yatein (Fig. 2). Podophyllotoxin and 4′-demethylpodophyllotoxin exhibited almost equivalent proteasome activation effects to anthricin, although slightly inferior. On the other hand, yatein and hinokinin showed significantly reduced maximum proteasome activity compared with anthricin. These results indicate that (1) the presence or absence of a hydroxyl group at the 4-position and a methyl group at the 4′-position does not affect proteasome activation ability and that (2) the flexibility of the E-ring by opening C-ring hinders the compound's ability to activate the proteasome. Interestingly, 4-*O*-glycosylation rendered the proteasome activation ability almost ineffective, neither enhancing nor reducing the activity.

### 3.3 Anthricin directly interacts with the human 20S proteasome

The results from Fig. 1, Fig. 2 suggest that anthricin directly interacts with the proteasome. A pull-down assay was performed to confirm whether anthricin has binding activity to the proteasome (Fig. 3). The human 20S proteasome was mixed with biotinylation reagent, labeling the lysine residues on the proteasome surface with biotin. The biotinylated proteasomes (250 pmol) were immobilized on streptavidin magnetic beads and then reacted with anthricin (20 μM, 20,000 pmol). After washing, the solvent was replaced with methanol (200 μL) to denature the proteasome, elute the bound anthricin, and quantify it using LC-MS/MS. When anthricin was mixed with the proteasome-immobilized beads, a clear peak corresponding to anthricin was detected. The anthricin concentration in the eluent was 20 μM (20 pmol/μL), indicating that 4000 pmol of anthricin was eluted from the 20S proteasome-immobilized beads. Given that 250 pmol of proteasome was introduced into the pull-down assay, it suggests that there are at least 16 binding sites for anthricin on the 20S proteasome. Importantly, the peak corresponding to anthricin was below the detection limit when anthricin was mixed with streptavidin beads without proteasome immobilization. These results indicate that anthricin directly interacts with the proteasome.

### 3.4 Anthricin binds near the β1, β2, and β5 subunits and reduces the distance between the side chains of Thr^1^, Asp^17^, and Lys^33^ (the catalytic site of the proteasome)

**Fig. 4:**
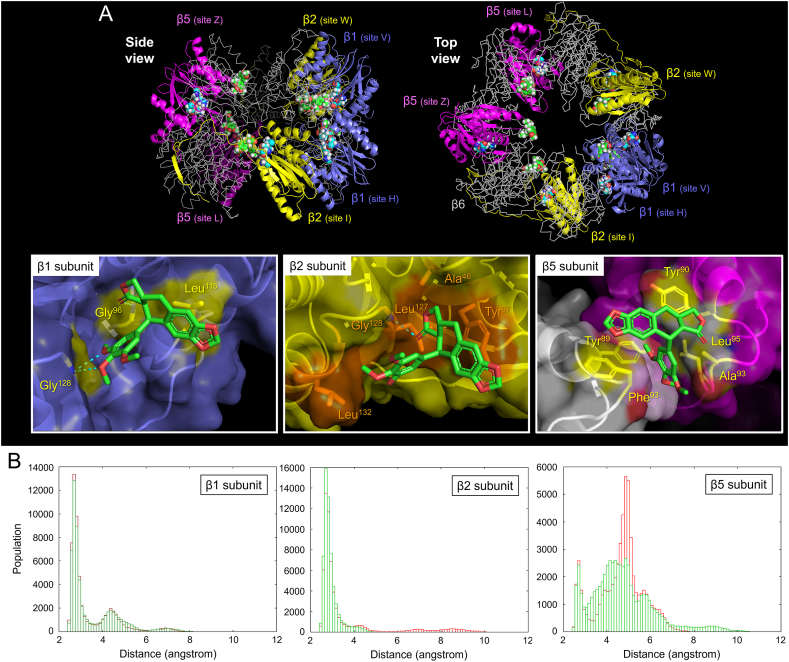
Molecular dynamics (MD) simulation of the interaction between anthricin and proteasome β-ring (A) MD simulations were performed based on the proteasome's crystal structure (*Saccharomyces cerevisiae*) complexed with K-7174, a proteasome inhibitor with similar structure to anthricin (PDB ID: 4EU2), by replacing K-7174 with anthricin. The representative complex structure after 200 ns simulation is shown. The β1, β2, and β5 subunits involved in enzyme activity are depicted in ribbons, whereas the other β-subunits are shown in gray lines. The three amino acid residues at the catalytic sites (Thr^1^, Asp^17^, and Lys^33^) of the β1, β2, and β5 subunits are shown as light blue spheres, and anthricin is depicted as green spheres. In the close-up view of the anthricin binding site, anthricin is shown as green sticks, whereas the interacting residues of the β1 subunit (Gly^96^, Gly^128^ and Leu^115^), the β2 subunit (Ala^46^, Tyr^97^, Leu^127^, Gly^128^ and Leu^132^), the β5 subunit (Tyr^90^, Ala^93^ and Leu^95^), and the β6 subunit (Tyr^89^ and Phe^93^) are shown as yellow or orange color sticks. (B) The three bar graphs plot the distances between the side chains of Thr^1^, Asp^17^, and Lys^33^ at the catalytic sites of the β1, β2, and β5 subunits during the 200 ns simulation. The red bars represent the apo form, whereas the green bars represent the proteasome bound with anthricin. (For interpretation of the references to color in this figure legend, the reader is referred to the Web version of this article.)

We performed MD simulations to infer the molecular mechanism by which anthricin enhances the function of the proteasome after binding to it. A notable chemical structural feature of anthricin is the trimethoxybenzyl group on the E-ring. Interestingly, K-7174, which contains two trimethoxybenzyl groups, has been reported as a proteasome-binding inhibitor. We hypothesized that anthricin binds to the proteasome in a similar manner to K-7174 [18]. We created a model of the anthricin/proteasome (two β-rings) complex through MD simulations by using the crystal structure of the K-7174/proteasome (two β-rings) complex (PDB ID: 4EU2) (Fig. 4). Three independent 200-ns simulations were conducted, and anthricin remained bound to the proteasome in all runs. Six anthricin compounds retained binding to the two β-rings. The number of binding sites for anthricin per 20S proteasome (comprising two α-rings and two β-rings) suggested by the pull-down test in Fig. 3 is 16, suggesting that anthricin may also have binding sites in the α-ring. Fig. 4A shows representative anthricin binding sites. In the β1 subunit, the oxygen of the D-ring of anthricin formed a hydrogen bond with the main-chain nitrogen of Gly^96^, while the A–C rings hydrophobically interacted with the side chain of Leu^115^, and the oxygens of the E-ring formed two hydrogen bonds with the main-chain nitrogen of Gly^128^. In the β2 subunit, the oxygen of the D-ring formed a hydrogen bond with the main-chain nitrogen of Gly^128^, while the A–D rings interacted with the hydrophobic surface formed by Ala^46^, Tyr^97^ and Leu^127^, and the E-ring hydrophobically interacted with the side chain of Leu^132^. In the β5 and β6 subunits, anthricin interacted to insert a trimethoxybenzyl group into the hydrophobic groove comprising Tyr^90^, Leu^95^, and Ala^93^ at β5 and Tyr^89^ and Phe^93^ at β6. These results support the findings from Fig. 3, demonstrating the direct binding of anthricin to the proteasome.

Next, we inferred the mechanism by which proteasome function is enhanced. The proteasome is a Thr-type protease, and the catalytic activity of Thr^1^, Asp^17^, and Lys^33^ is known to be critically important [22]. According to Huber et al., the hydroxyl group of Thr^1^, deprotonated by Lys^33^, nucleophilically attacks and hydrolyzes the peptide bond of the substrate peptide, and then Asp^17^ acts on Lys^33^ to protonate the hydroxyl group of Thr^1^, returning it to its steady state. The orientation of these residues is strictly conserved in the β1, β2, and β5 subunits of the proteasome, and the distance between these residues is crucial. Therefore, the distances between the Thr^1^, Asp^17^, and Lys^33^ residues in the β1, β2, and β5 subunits during MD simulations of the anthricin-bound proteasome and apo-proteasome were extracted and compared (Fig. 4B). However, only Lys^33^ in the β5 subunit was replaced by Arg in the crystal structure of the K-7174 and proteasome complex (PDB ID: 4EU2) [18]. Thus, the δ-position NH of the Arg side chain was tracked as if it were the Lys side chain. The results showed no significant difference between the anthricin-bound and apo-proteasomes for β1. However, for β2, the distance between the Thr^1^, Asp^17^, and Lys^33^ residues in the anthricin-bound proteasome was significantly shorter than that in the apo-proteasome. For β5, both populations with distances between Thr^1^, Asp^17^, and Lys^33^ residues of 3–4.5 Å and those with distances between 6.8 and 12 Å increased, but the population increasing rate was greater in the former. Considering the net difference from the apo form per angstrom, the 3–4.5 Å population increased by 6102/Å and the 6.8–12 Å population by 745/Å, with the former showing an 8.2-fold greater increase. Namely, in the overall population, shorter distances between the catalytic residues have increased. These findings suggest that the Thr^1^, Asp^17^, and Lys^33^ residues, which are crucial for catalytic activity, are closer to each other in the anthricin-bound proteasome than in the apo form, thereby facilitating enzyme activity. The observation that the distance between the three residues in β2 and β5 changed in the MD simulations correlates well with the fact that anthricin's proteasome-activating effect is prominent in the trypsin-like and chymotrypsin-like activities involving β2 and β5 (Fig. 1).

## 4 Discussion

Proteasomes play a pivotal role in maintaining intracellular homeostasis and protecting the central nervous system. Some studies suggest that high proteasome activity is correlated with longevity [23]. Recently, proteasomes have also gained attention from a drug discovery perspective, particularly in the context of protein degradation inducers. Therefore, compounds that enhance proteasome activity are expected to contribute not only to the health and longevity of organisms but also to drug development. In this study, we showed for the first time that anthricin, a natural product, directly interacts with the proteasome and enhances its activity by combining biochemical experimental methods and in silico MD simulations.

The present study reveals several interesting aspects of anthricin's proteasome-activating effects. The first aspect is the potency of the proteasome-activating effect shown in Fig. 1. Several natural and synthetic compounds are known as proteasome activators. However, anthricin stands out because of its potent activity. For example, anthricin demonstrates activity in the 100 nM range, as shown in this study, whereas the proteasome-activating effect of chlorpromazine occurs in the μM range. The second aspect is the efficacy of the proteasome-activating effect shown in Fig. 2. Especially in chymotrypsin-like activity, the proteasome activity was increased by about fivefold in the presence of 100 nM anthricin. Interestingly, if anthricin is considered as a full agonist, its analog yatein acts as a partial agonist, and podophyllotoxin-4-*O*-glucoside shows no proteasome-activating effect. The difference between anthricin and yatein might be attributed to the entropic loss during binding because of the E-ring's flexibility. On the other hand, the difference between anthricin and podophyllotoxin-4-*O*-glucoside is harder to explain. Although glucosylation might reduce the permeability of the cell membrane, the glycosylated compound of 4′-demethylpodophyllotoxin at the 4 position is used as the anticancer drug etoposide, which works through a mechanism involving the cleavage of intracellular DNA strands [24]. The glucoside group may be a steric hindrance to binding to the proteasome. The third aspect is that anthricin enhances activity in the following order: chymotrypsin-like activity (β5) > caspase-like activity (β1) > trypsin-like activity (β2). From the results of MD simulations shown in Fig. 4, anthricin interacts in close proximity to the catalytic sites (Thr^1^, Asp^17^, and Lys^33^) of β1, β2, and β5. The interaction region of anthricin with the β1 and β2 subunits appears shallow and flat, which may make it less suitable for stable binding. In contrast, the interaction region in the β5 subunit forms a groove, suggesting it is better suited for binding compared to that of the β1 and β2 subunits. The binding of anthricin to β5, more than to β1 or β2, is thought to restrict protein movement, thereby increasing folding and stabilizing the distances between the catalytic residues. However, the detailed reason behind this mechanism remains unclear.

The MD simulation of anthricin and proteasome was based on the co-crystal structure of the K-7174 and proteasome complex (PDB ID: 4EU2) [18]. The structural feature of anthricin (trimethoxybenzyl group) matches with that of K-7174. However, while the former is a proteasome activator, the latter is a proteasome inhibitor. K-7174 has two trimethoxybenzyl groups connected via a linker structure, which may allow it to bridge two binding sites on the proteasome and inhibit its function by restricting its movement. Although this study elucidated one part of the molecular mechanism behind the proteasome-activating action of lignans, including anthricin, more detailed studies, especially the analysis of the co-crystal structure of anthricin or its analogs and proteasomes, will be needed in the future.

Although anthricin enhances proteasome activity, it is difficult to apply it as a pharmaceutical for this purpose. As mentioned previously, the glycosylated derivative of anthricin (i.e., 4′-demethylpodophyllotoxin) is used as the anticancer drug etoposide. Given that anthricin is likely to exhibit cytotoxicity at high concentrations, its application might lead to significant side effects. Based on the findings of this study, structural modifications of anthricin could lead to the design of new proteasome activators with fewer side effects in the future. These activators could contribute to drug discovery, offering neuroprotection or enhancing protein degradation-inducing drugs.

## Author contributions

KS and TH provided the conception and design of the study. KS conducted proteasome assay and pull-down assay. RF conducted measurement of compound by LC-MS/MS. EKA evaluated mRNA expression assay. TH conducted MD simulation. KS and TH wrote the draft of the manuscript. All authors received and approved the final manuscript for submission.

## Funding

This study was conducted without financial support from any external organizations.

## Declaration of generative AI in scientific writing

The authors declare that no generative AI or AI-assisted technologies were used in the creation of this manuscript.

## Declaration of competing interest

KS, RF and EKA is a full-time employees of Ichimaru Pharcos Co. Ltd. TH declare that there are no competing interests associated with this manuscript.

## Data Availability

Data will be made available on request.
